# Weight perception and risk of non-communicable diseases among women: A cross-sectional study in Ghana

**DOI:** 10.1371/journal.pgph.0004931

**Published:** 2025-08-04

**Authors:** Enoch Sam Sakyi Asiedu, Gloria Ethel Otoo, Agartha Ohemeng

**Affiliations:** Department of Nutrition and Food Science, School of Biological Sciences, University of Ghana, Legon, Accra, Ghana; Universiti Malaya, MALAYSIA

## Abstract

Objective measures of overweight/obesity have shown consistent association with other key non-communicable diseases (NCDs) such as type II diabetes and hypertension, but there is a gap in knowledge about the role of how people’s perceived weight in the aetiology of these diseases, especially in the African context. This study investigated the relationship between weight perception and NCD risk factors among women living in an urban setting in Ghana. A cross-sectional design was employed to recruit 378 female adults aged 18–65 years in the Accra Metropolis using convenience sampling method. Weight perception was assessed using the Feel-weight-status minus Actual-weight-status Index. Obesity was diagnosed using BMI based on the World Health Organization cut-offs. Elevated blood sugar level and pressure were assessed through a single random blood sugar test and multiple blood pressure readings, respectively. Regression models were used to determine associations between weight perception and NCD risk factors, while controlling for potential confounders. Approximately 80% of overweight participants and 90% of obese participants underestimated their weight. The proportion at risk of central obesity, elevated blood pressure, and elevated blood glucose level were 49.5%, 29.1%, and 6.6%, respectively. Age was associated with higher odds of elevated blood glucose levels (OR = 1.049, 95% CI: 1.026 - 1.073, p < 0.0001), elevated blood pressure (OR = 1.051, 95% CI: 1.031 - 1.072, p < 0.0001), and overweight/obesity (OR = 1.065, 95% CI: 1.042 – 0.089, p < 0.0001). Accurate weight perception was associated with lower odds of overweight/obesity (OR = 0.069, 95% CI: 0.038, 0.126, p < 0.0001), but was not significantly associated with elevated blood pressure and blood glucose levels. Accurate weight perception was negatively associated with obesity among the study participants. Public health education is needed to promote accurate weight perception among women as this may help to address objective measures of overweight/obesity and mitigate NCD risk in this sub-population.

## Introduction

### Prevalence of NCDs (obesity, hypertension and diabetes)

Non-communicable diseases (NCDs), which include conditions such as obesity, diabetes mellitus, cardiovascular diseases, and hypertension, have emerged as leading contributors to global morbidity and mortality rates [1]. According to the World Health Organization (WHO) 2024 report, NCDs account for over 70% of global deaths, with low- and middle-income countries (LMICs) bearing approximately three-quarters of these fatalities [2]. The recent global prevalence of overweight and obesity among adults is 16% and 43% respectively [3]. In Sub-Saharan Africa, including Ghana, urbanisation and the accompanying dietary and lifestyle transitions are driving the surge in obesity rates. The prevalence of obesity is increasing among South African women, and it is expected to hit 47.7% by the end of 2025 [4] and the prevalence of overweight/obesity in Nigerian women is reported to be almost 50% [5].The Global Obesity Observatory revealed that obesity rates in Ghana have more than doubled in recent decades, with a national prevalence of 15.0% in 2015 compared to 6.4% in 2004 [6]. Obesity prevalence among women in the country has been reported to be around 35.4%, a significant increase from earlier decades [7]. This rise in obesity poses a major public health concern given its strong link to the development of other NCDs. In Ghana, NCDs account for 43% of all deaths with diabetes and hypertension being the major contributors to this mortality rate [8]. NCDs are characterised by their chronic nature and their associations with modifiable lifestyle factors, including poor diet, physical inactivity, and tobacco and alcohol use [9].

Among the diverse risk factors driving the global NCD epidemic, obesity which is an established NCD, doubles up as a major risk factor for other NCDs such as type II diabetes, hypertension, cardiovascular diseases, and certain cancers [10] These conditions are not only leading causes of death but also major contributors to disability-adjusted life years (DALYs) [11]. The prevalence of hypertension in particular is high in some African countries, including the Democratic Republic of Congo (26%), Botswana (33%), and Angola (34%) [12–14]. Generally, the prevalence of hypertension among women across the continent is approximately 48% [15]. According to the WHO, the prevalence of diabetes among adults in Africa has increased from 6.4% in 1990 to 10.5% in 2022 [16]. In Ghana, the shift from infectious diseases to NCDs as the predominant health concern has placed additional strain on healthcare resources [17]. The most prevalent NCDs in Ghana are obesity, diabetes mellitus, and hypertension [8,17].

### Weight perception and its importance

Weight perception is defined as the subjective assessment of an individual’s body weight, which may or may not align with objective measures like body mass index (BMI) based on the WHO cut-offs [18]. Weight perception can significantly influence health behaviours, including diet, physical activity, and participation in weight management programs [19]. According to the Health Belief Model (HBM), individuals’ engagement in health-related behaviour is influenced by their perceived susceptibility to health problems, perceived severity of those problems, perceived benefits of action, and perceived barriers to action [20]. This means individuals who underestimate their weight may perceive a lower susceptibility to obesity and its related health risks and will be less likely to adopt health-promoting behaviours. Conversely, those who overestimate their weight may perceive high susceptibility and resort to harmful practices like extreme dieting or unregulated weight-loss supplements [21]. These behaviours are often driven by the belief that short-term solutions will be effective and that behavioural change requires minimal effort. Studies indicate that overweight/obese individuals often underestimate their weight status [22]. This widespread underestimation presents significant public health challenges, as it can hinder effective weight management and worsen the obesity epidemic. Few studies indicate that inaccurate weight perception increases the risk of Type II diabetes and its complications [23] and hypertension [8].

### Association between weight perception and NCDs

Thus, weight perception may be linked to some NCDs and associated risk factors. There is, however, a lack of similar information from the African context. Weight perception has also been shown to have a bidirectional relationship with health behaviours, influencing not only dietary and physical activity patterns but also mental health and overall well-being [24]. Weight perception is in turn shaped by factors such as body image, cultural norms, socioeconomic status, and access to health information [25].

Concerns about weight are generally observed more among women because they are concerned about their body size and appearance [26] influenced by cultural and societal factors. For instance, Okop et al. [27] revealed that 79% of overweight individuals and 85% of obese women underestimated their weight. Again, 61.2% of women in Nigeria [28], 41% of women in Mauritius [29], and 32.8% of respondents in a study in Tanzania misperceived their weight [30]. In many African societies, including Ghana, fuller body sizes are often seen as symbols of beauty, wealth, and social status [31]. This cultural norm can lead overweight and obese individuals to underestimate their weight.

Observations in Ghana show an increasing trend of NCDs among women, particularly in the urban settings [5,8,32]. This demographic goes through significant dietary and lifestyle changes, alongside weight misperceptions, which can increase their susceptibility to these conditions. While studies have sought to investigate various potential objective risk factors, there is an obvious lack of information on the role of a subjective factor such as one’s weight perception in the aetiology of common NCDs in Ghana. Therefore, this study hypothesised that women who underestimate their weight status will have a higher risk of obesity, hypertension, and type 2 diabetes, compared to those who accurately perceive their weight. Such information can inform targeted interventions and health promotion strategies tailored to reduce the burden of NCDs. This study aligns with the broader global public health agenda (Sustainable Development Goal 3) which emphasises the importance of reducing the burden of NCDs and promoting “Good health and well-being” for all.

## Methods

### Ethics statement

Ethical clearance for this study was obtained from the Ghana Health Service Ethics Review Committee (GHS-ERC: 029/01/24) and the Ethical Committee of the College of Basic and Applied Sciences (ECBAS 029/23–24), University of Ghana. Permission was also obtained from the mall’s management and the association heads of the open markets before their facilities were used for the study. The participants who agreed to take part in the study either signed or thumb-printed a consent form before data collection began.

### Sampling procedure

Data collection started on February 27, 2024 and ended on May 13, 2024. One shopping mall and three open markets were randomly selected from a list of shopping malls and open markets in the Accra metropolis. Participants for the study were selected from the study locations using a convenience sampling method, which is a non-probability sampling technique where participants are selected based on their availability and willingness to take part in the study [33]. This approach was considered suitable given the exploratory nature of the study, which sought to generate initial insights into weight perception and related health outcomes among women in Ghana. Although convenience sampling may introduce selection bias and limit the generalizability of findings, the use of markets and shopping mall enabled the inclusion of a socioeconomically diverse sample within the practical constraints of time and resources. Potential confounding factors such as age and socioeconomic status were measured and statistically controlled for in the analysis to enhance the internal validity of the findings. When the research team arrived at each location, research assistants approached women (18–65 years old), explained the purpose of the study, and invited them to participate. Those who were interested and provided their consent were then included in the study. Pregnant women and women with disabilities or challenges that could have negatively affected anthropometric measurements were excluded from the study.

### Data collection

At each study location, the research team set up a stationary point where all the data collection instruments were administered. The participants who agreed to participate in the study were directed to the stationary point where they either signed or thumb-printed a consent form. Then participants’ sociodemographic and weight perception information were collected, and anthropometric measurements were taken. Blood pressure measurements and blood samples for glucose sugar levels were also taken.

### Anthropometric assessment

Anthropometric measurements constituted participants’ height, weight, hip and waist circumferences. All measurements were conducted according to standard protocol [34], with participants wearing clothing. However, participants were required to remove heavy garments like jackets and sweaters, shoes or sandals, belts, watches, and other wrist accessories, and to empty their pockets before the measurements. Weight measurements were done using a digital scale (BWB-800, TANITA, Japan) and values were recorded in kilograms to the nearest 0.1 kilograms. For height measurement, a well-mounted and calibrated portable stadiometer (ShorrBoard, weigh and measure LLC, USA) was used and height was recorded to the nearest 0.1 centimetres. All measurements were taken three times, and the average was used in the subsequent calculations. The weight and height measurements were inputted into Quetelet’s equation [weight (kg)/height^2^(m^2^)] to calculate the BMI. Participants’ BMI was compared with the WHO cut-offs [25] where; BMI less than 18.5 kg/m^2^ was classified as underweight, from 18.5 kg/m^2^ to 24.9 kg/m^2^ was classified as normal weight, BMI from 25 kg/m^2^ to 29.9 kg/m^2^ was classified as overweight, and a BMI greater than 29.9 kg/m^2^ was classified as obese.

Waist and hip circumferences were according to international specifications [35], to the nearest 0.1 cm and used to compute the waist-to-hip ratio (WHR). The WHR was used to evaluate the central obesity status of the participants. For women, a value equal to or less than 0.85 was considered normal, and a value higher was considered abnormal according to the WHO cut-offs [36].

### Blood pressure measurement

An automatic arm sphygmomanometer device (SBM 52, Sanitas, Germany) was used to measure the blood pressure of all participants. The measurement was done on the upper left arm while the subject sat with legs uncrossed, the arm was supported at the height of the heart, and the arm was wrapped in a cuff that was appropriate for the arm size. The blood pressure measurement was taken after each participant had rested for at least 10 minutes. Measurements were repeated at 5 minutes intervals and the average systolic blood pressure and diastolic blood pressure were recorded. Elevated blood pressure leading to hypertension was defined as systolic pressure levels ≥140 mmHg and/or diastolic levels ≥90 mmHg according to the 2018 European Society of Cardiology/European Society of Hypertension Guidelines [37].

### Random blood glucose test

A digital glucometer (Safe-Accu 2, Sinocare, China) was used to measure a single random blood glucose level. Participants were instructed to wash and dry their hands thoroughly to ensure accurate results. For each test, a new test strip and lancet were used. The left thumb was prepared by cleansing with an alcohol swab, followed by a controlled prick to collect a small blood sample. This blood was applied to the test strip according to the manufacturer’s guidelines, allowing for automatic absorption. The glucometer was checked and adjusted periodically to maintain accurate and reliable measurements. Special care was taken to minimise contamination and ensure sterility during sample collection. After processing, the results were recorded promptly, and participants with elevated readings were advised to seek further medical evaluation. A random blood glucose level of 11.1 mmol/L (≥200 mg/dL) or higher indicated elevated blood glucose, a risk factor for type II diabetes mellitus [16]

### Measurement of weight perception

The Pulver’s figure rating scale was used to assess participants’ feel-weight status [38]. It provides a simple and visual way to classify individuals based on their perceived body weight. The scale has nine (9) silhouettes, and each silhouette is associated with a specific score that corresponds to a particular body weight status. The breakdown of the scales was as follows: underweight (silhouettes 1 and 2, scored as 1), normal weight (silhouettes 3, 4, and 5, scored as 2), overweight (silhouettes 6 and 7, scored as 3), and obese (silhouettes 8 and 9, scored as 4 [38,39]. The Feel-weight-status minus Actual-weight-status Index (FAI) was used to determine participants’ weight perception. FAI is an indicator used to determine how accurate an individual’s perceived weight is based on his or her actual weight status. The actual weight (BMI) of participants was based on the WHO’s classification: underweight (BMI less than 18.5 kg/m^2^, scored as 1), normal weight (BMI from 18.5 kg/m^2^ to 24.9 kg/m^2^, scored as 2), overweight (BMI from 25 kg/m^2^ to 29.9 kg/m^2^, scored as 3), and obese (BMI greater than 29.9 kg/m^2^, scored as 4). FAI was calculated by subtracting the actual weight (BMI) score from the feel-weight score. A zero (0) FAI score meant that the individual had accurate weight perception. A positive score indicates that the participants perceived themselves to be heavier than they were while a negative score indicates that the participants perceived themselves to be lighter than they were.

### Data analysis

The data was entered and cleaned using Microsoft Excel 2019. The statistical analysis was done using Statistical Package for Social Sciences (SPSS) version 27. All analyses were done at a confidence level of 95% and a margin of error of 5%. Continuous data were presented in means (X̄) and standard deviations (SD) and categorical data were presented as frequencies (n), and percentages (%). Descriptive statistics were used to analyse data on the sociodemographic characteristics, nutritional status, weight perception, elevated blood glucose, and elevated blood pressure of the participants. Binary logistic regression models were used to determine the relationship between weight perception and the risk of NCDs among the participants. Variables that were statistically significant at p-value = 0.05 in the bivariate analyses were added to the final models as covariates. Even though parity was significant at the unadjusted model, it was not added to the final models because it correlated with age [correlation coefficient (r) = 0.639]. Age was chosen over parity because it is considered a better determinant of weight perception in the existing literature [40].

## Results

The average age of the study respondents was 42.1 years (± 13.2). The age distribution revealed that more than half (54.2%) fell within the range of 26–50 years, as detailed in Table 1. Majority of respondents were traders (70.4%), while only a small fraction (2.6%) reported being unemployed. The average reproductive history indicated that respondents had experienced an average of 2.0 (± 2) live births. The income distribution revealed that 54.8% of respondents earned less than GH₵ 1000.00 monthly. while nearly a quarter (23.0%) earned GH₵ 2,000.00 or more monthly.

**Table 1:** Socio-demographic characteristics of respondents.

Variable	Frequency (N)	Percentage (%)	Mean ± SD
**Age, years**			42.1 ± 13.2
**Education Status**			
None	79	20.9	
Basic	142	37.6	
Secondary	126	33.3	
Above	31	8.2	
**Occupation**			
Professional/office worker	78	20.6	
Trader	266	70.4	
Other	24	6.4	
Unemployed	10	2.6	
**Marital status**			
Never married	142	37.6	
Married/cohabiting	194	51.3	
Previously married	42	11.1	
**Parity**			2.0 ± 2
**Religion**			
Christian	340	89.9	
Muslim	38	10.1	
**Income groups**			
< 1000	207	54.8	
1000 – 1999	84	22.2	
≥ 2000	87	23.0	

The average BMI of the participants was 28.8 kg/m² (± 6.1), and two-thirds (68%) falling within the overweight/obesity category (Table 2). About half (49.5%) were classified as having a high waist-to-hip ratio. Most of the study women (70.1%) had inaccurate perception about their weight status, with majority of them perceiving themselves to be lighter than their actual weight. Conversely, less than a third exhibited accurate weight perception, reflected by an FAI score of zero.

**Table 2:** Nutritional status (BMI and WHR) and weight perception of respondents.

Variable	Frequency (N)	Percentage (%)	Mean ± SD
**BMI, kg/m** ^**2**^			28.8 ± 6.1
**BMI category**			
Underweight	6	1.6	
Normal	115	30.4	
Overweight	113	29.9	
Obese	144	38.1	
**Waist-to-Hip Ratio**			0.86 ± 0.12
**Waist-to-Hip Ratio category**			
Normal	191	50.5	
High	187	49.5	
**Weight perception category**			
Accurate	113	29.9	
Inaccurate (lighter)	9	2.4	
Inaccurate (heavier)	256	67.7	

**Fig 1:**
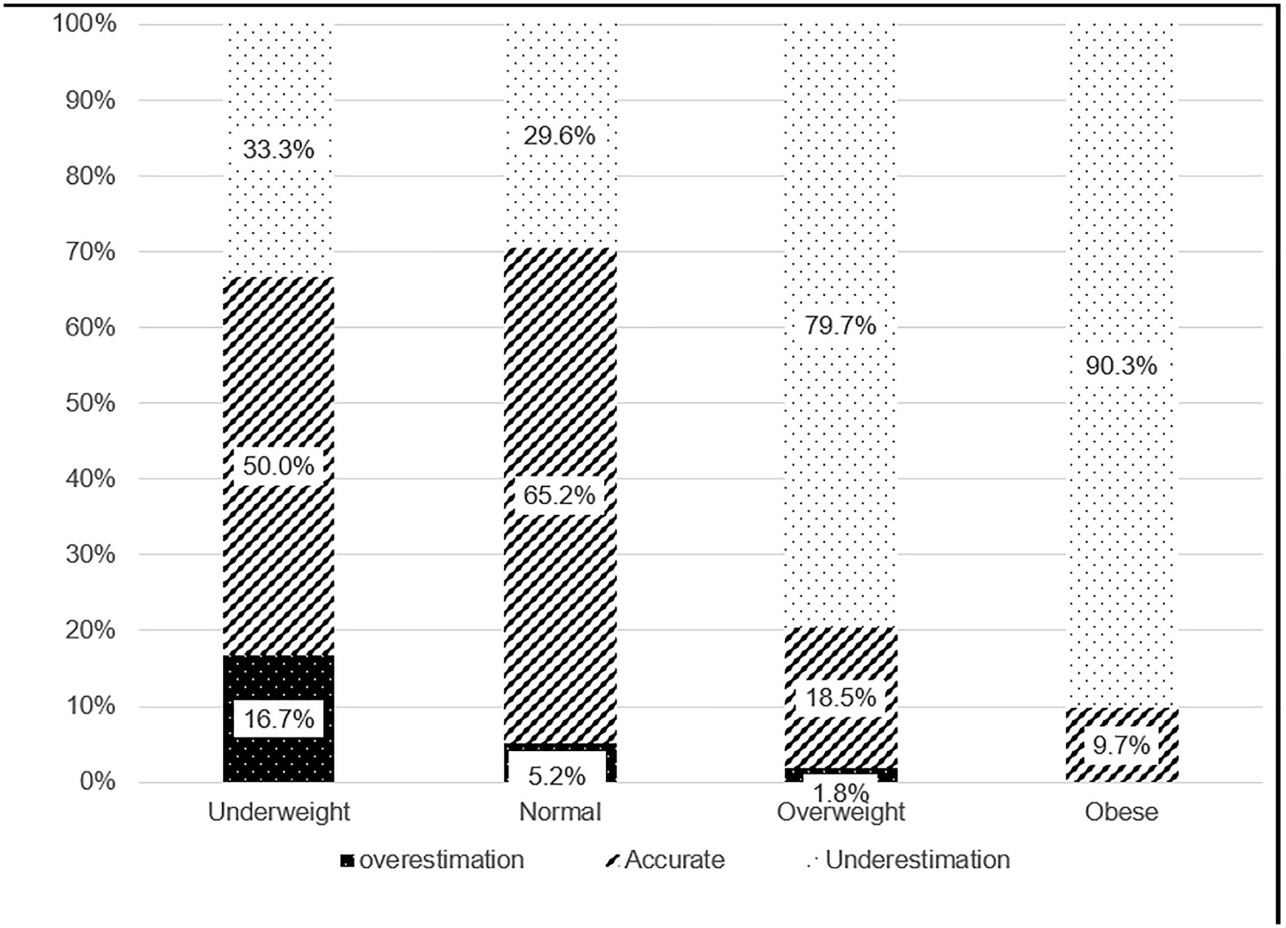
Weight perception of respondents based on their nutritional status.

A significant proportion of overweight (80%) and obese (90%) participants underestimated their weight status (Fig 1). Conversely, 65% of participants with a healthy body weight based on BMI accurately perceived their weight.

**Fig 2:**
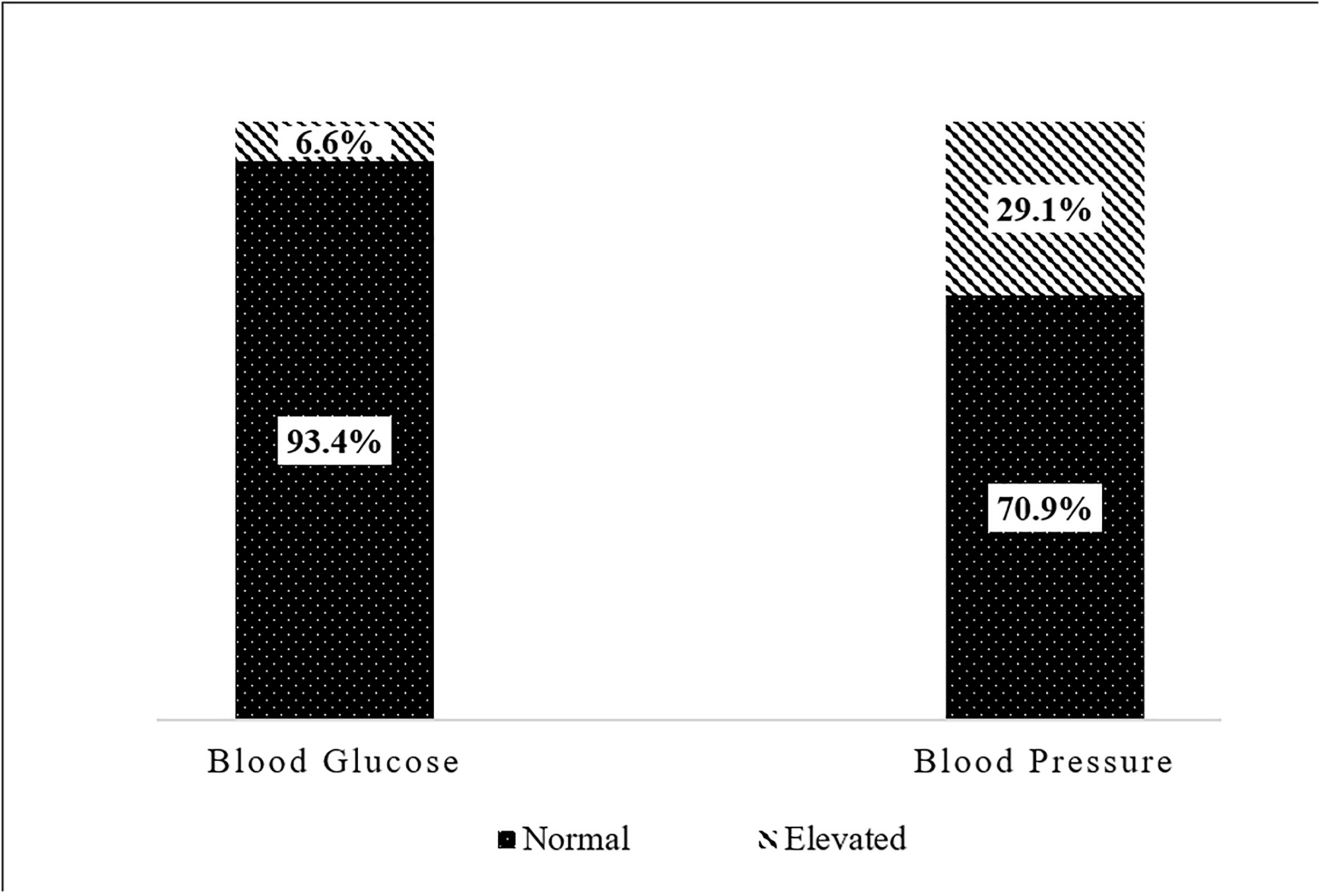
Prevalence of elevated blood glucose and elevated blood pressure of participants.

Fig 2 reveals that 6.6% of respondents recorded elevated blood glucose levels, while the majority (93.4%) maintained normal blood glucose levels. The average random blood sugar level of the participants was 6.9 ± 3.0 mmol/L. The average blood pressure among participants was measured at 125/80 mmHg, and more than two-thirds (71%) had normal blood pressure levels.

Bivariate analysis showed that age, education, occupation, income, and weight perception (S1 Table) were independently associated with nutritional status represented by BMI categories. After accounting for covariates, women with accurate weight perception were approximately 93% less likely (OR = 0.069, 95% CI: 0.038 - 0.126, p < 0.0001) to be overweight/obese (Table 3). However, weight perception was not associated with elevated blood glucose (OR = 0.657, 95% CI: 0.354 – 1.218, p = 0.182) or blood pressure levels (OR = 0.977, 95% CI: 0.579 – 1.648, p = 0.929). Additionally, a unit increase in age increased respondents’ likelihood of being overweight/obese by 5.5% (OR = 1.055, 95% CI: 1.030 – 1.081, p < 0.0001), likelihood of having elevated blood glucose by 4.9% (OR = 1.049, 95% CI: 1.026- 1.073, p < 0.0001), and likelihood of having elevated blood pressure by 5.1% (OR = 1.051, 95% CI: 1.031- 1.072, p = 0.0001). Compared to those who reported earning at least GH₵ 2,000.00 per month, women who earned a monthly income less than GH₵ 1,000.00 were approximately 47% less likely to have elevated blood glucose (OR = 0.533, 95% CI: 0.299 – 0.951, p = 0.033). Those who earned a monthly income from GH₵ 1,000.00 to GH₵ 1,999.00 were 73% less likely to have elevated blood glucose (OR = 0.261, 95% CI: 0.113 – 0.603, p = 0.002) and 50% less likely to have elevated blood pressure as compared to participants who were earning GH₵ 2,000.00 and above (OR = 0.503, 95% CI: 0.290 – 0.872, p = 0.014).

**Table 3:** Association between weight perception and NCD risk factors among the study participants, based on binary logistic regression.

Variables	^a^ Overweight/obesity	^b^ Elevated blood glucose	^c^ Elevated blood pressure
	OR (95% Cl)	OR (95% Cl)	OR (95% Cl)
**Age**	1.055(1.030, 1.081)^*^	1.049(1.026, 1.073)^*^	1.051(1.031, 1.072)^*^
**Income category**			
Ref= ≥ 2000	1.000	1.000	1.000
< 1000	0.701(0.347, 1.417)	0.533(0.299, 0.951)^*^	0.600(0.309, 1.165)
1000 - 1999	1.048(0.453, 2.423)	0.261(0.113, 0.603)^*^	0.503(0.290, 0.872)^*^
**Weight perception**			
Ref = Inaccurate	1.000	1.000	1.000
Accurate	0.069(0.038, 0.126)^*^	0.657(0.354, 1.218)	0.977(0.579, 1.648)

^a^Refers to body mass index which indicates overweight/obese, i.e., BMI ≥ 25.0 kg/m^2^

^b^Refers to random blood glucose which indicates elevated blood glucose, i.e., RBS ≥ 11.1 mmol/L

^c^Refers to blood pressure which indicates elevated blood pressure, i.e., BP ≥ 140/90 mmHg

Amount in Ghana Cedis (GH₵), represents reported monthly income

Ref. = Reference category,

* Statistically significant at p < 0.05

## Discussion

### Nutritional status of respondents

Almost half (49.5%) of the women surveyed were centrally obese, a condition closely linked to cardiovascular diseases and metabolic syndromes. Central obesity, characterised by excess visceral fat around the abdomen, is associated with insulin resistance, dyslipidaemia, and hypertension. Thereby increasing the risk of cardiovascular disease and other non-communicable diseases [41]. The high prevalence of overweight (29.9%) and obesity (38.1%) among the respondents mirrors national trends, indicating a rise in overweight/obesity rates across the country [5].

Approximately 29% of respondents were found to be at risk of hypertension, consistent with other findings from other urban settings in the country [41,42]. These studies reported notably higher prevalence than the 13% reported in the 2014 Ghana Demographic and Health Survey [43]. This pattern extends across sub-Saharan Africa, where hypertension affects between 20% to 30% of adults [44]. Urbanisation-related lifestyle changes, such as the shift from traditional diets and decreased physical activity, are major factors driving this increase in hypertension [44]. On the other hand, elevated blood glucose, a precursor to diabetes, was found in less than 10% of the study participants. Previous studies have reported similar findings, with Gatimu et.al. [45] noting a national prevalence of diabetes of 3.95% among older adults with a higher rate in women compared with men. This is at the lower end of what has been observed across the African region, where Type II diabetes affects 4.5% to 15% of adults, particularly in urban areas [46,47]. The coexistence of elevated blood pressure and elevated blood glucose levels in this population is alarming given its association with Metabolic Syndrome (MetS), which has emerged as a critical concern due to its association with various NCDs. For instance, recent studies indicate that the prevalence of MetS in urban African communities is high, with figures reaching up to 58.4% in certain populations [48,49]

### Weight perception of respondents

A significant proportion of overweight and obese women in this study underestimated their weight, with about 80% of overweight and 90% of obese participants misjudging their weight and perceived themselves to weigh less than they did. Cultural norms likely influence this in Ghana, where fuller body sizes are often seen as signs of beauty and wealth [50,51]. Respondents who accurately perceived their weight were approximately 93% less likely to be overweight or obese after accounting for potential confounders. While this association does not imply causality, it underscores the potential role of accurate weight perception in supporting healthier outcomes. Accurate weight perception may be essential for effective health management, as it can directly influence individuals’ motivation to engage in health-promoting behaviours [22]. Globally, studies have shown that individuals with accurate weight perceptions are more likely to adopt health-promoting behaviours, such as increasing physical activity and improving dietary patterns [52,53]. This study contributes to understanding whether global patterns that link weight perception to behaviour and health outcomes apply in the Ghanaian context. The findings suggest that culturally tailored interventions promoting weight awareness could support national efforts to address obesity and related non-communicable diseases.

### Relationship between weight perception and NCDs risks

The study noted no significant association between weight perception and elevated blood pressure or blood glucose levels, contrary to prior research that indicated that individuals who misperceive their weight were at a higher risk of elevated blood glucose levels and blood pressure [23,54]. The differences in the findings may partly be due to differences in study populations, research designs, and cultural perceptions of body image. The current study’s cross-sectional design and focus on adult women in an urban Ghanaian context limit direct comparability to longitudinal studies in other settings. Future research involving diverse populations and longitudinal methods may help clarify the nature of these associations.

Age emerged as a significant predictor of overweight/obesity, elevated blood pressure, and elevated blood glucose levels among the women surveyed. These findings are consistent with existing evidence that links ageing with physiological changes such as increased arterial stiffness, reduced insulin sensitivity, and changes in body composition that increase the risk of NCDs [3,15,44]. Additionally, lifestyle shifts that occur with age, including reductions in physical activity and dietary changes, may contribute to this trend [55].

Income was significantly associated with blood glucose levels and blood pressure. The findings challenge the conventional belief that higher income is universally associated with better health outcomes due to enhanced access to healthcare and nutritious foods. Middle income represents a socioeconomic “sweet spot”, where financial means support health prioritization without exposure to the unhealthy lifestyle choices often seen in wealthier or poorer populations [56]. Conversely, higher income earners may be linked with lifestyle factors that increase NCD risk, such as frequent consumption of processed foods, dining out, and a more sedentary occupation due to less physically demanding work environments [57]. Consequently, this demographic may face an elevated risk of these conditions despite their financial capacity to access health-promoting resources. Conversely, low-income individuals often struggle to obtain nutritious foods and may rely on cheaper energy-dense options that can exacerbate their risk of NCDs.

Overall, this study revealed the growing burden of obesity and its related NCDs among women living in urban Ghana and showed that inaccurate body image perception is associated with an increased risk of overweight/obesity among the women. However, the study has some limitations. The use of a cross-sectional design limits the ability to establish causation between observed risk factors and health outcomes. Longitudinal studies, scale validation, or intervention trials would provide deeper insights into how these risk factors evolve. The study was also conducted in an urban setting, meaning the findings may not be entirely generalisable to rural populations, where different dietary habits and levels of physical activity may yield different results. Lastly, while income was found to be a significant predictor of certain health outcomes, the study did not account for other socio-economic determinants, such as housing and living conditions and healthcare accessibility, which could influence these relationships.

## Conclusion

The study revealed that most overweight and obese participants underestimated their weight status and that inaccurate perception of one’s weight was associated with an increased risk of being overweight or obese, highlighting the possible role of accurate weight perception in mitigating the risk of obesity and its related health problems. This underscores the need for targeted public health strategies that incorporate culturally sensitive weight perception components into existing health education programs. For example, community-based initiatives and media campaigns that promote realistic and health-oriented body image could support individuals in developing a more accurate understanding of their weight status. Middle-income earners demonstrated lower risks of elevated blood glucose and elevated blood pressure, suggesting that socioeconomic factors are key factors in health outcomes. Interventions that target health equity, such as expanding access to affordable, nutritious foods and preventive healthcare services across income groups. Furthermore, integrating routine weight and metabolic screening into primary care settings, especially in urban areas, could support early diagnosis of NCD risks and help tailor interventions more effectively. These findings point to the need for a multi-level approach to NCD prevention that combines individual awareness with structural and policy-level efforts. Future research should explore how targeted health messaging, socioeconomic support systems, and culturally grounded interventions can be leveraged to improve metabolic health outcomes in Ghana and similar urban African settings.

## Supporting information

S1 Table Bivariate association between sociodemographic characteristics and NCD risks (overweight/obesity, elevated blood glucose, and elevated blood pressure) among the study participants. (DOCX)

S1 Data Weight perception and NCD risk. (XLSX)
