# Supplementary material for: Weight perception and risk of non-communicable diseases among women: A cross-sectional study in Ghana
Source: PLOS Glob Public Health. 2025 Aug 4;5(8):e0004931. doi: 10.1371/journal.pgph.0004931 (PMC12321078; doi:10.1371/journal.pgph.0004931)
Supplement: S1 Table — (DOCX) [file pgph.0004931.s001.docx]

**Relationship between NCD risks and Sociodemographic Characteristics**

Supplementary Table 1 (S1 Table) presents the bivariate associations between sociodemographic characteristics and the NCD risk factors (overweight/obesity, elevated blood glucose, and elevated blood pressure) among study participants. Age was significantly associated with all three NCD risk factors. The odds ratios indicate that for each year increase in age, the likelihood of being overweight or obese increased by 6.1% (OR = 1.061, 95% CI: 1.042 – 1.081, p=0.0001), while the odds of elevated blood glucose rose by 5.2% (OR = 1.052, 95% CI: 1.030 – 1.074, p=0.0001) and the risk of elevated blood pressure also increased by 5.3% (OR = 1.053, 95% CI: 1.033 – 1.073, p=0.0001). Parity was also significantly associated with an increased risk for all three outcomes. Specifically, the odds of overweight/obesity increased by 43.2% (OR = 1.432, 95% CI: 1.242 – 1.651, p=0.0001), while the likelihood of elevated blood glucose and elevated blood pressure increased by 29.4% (OR = 1.294, 95% CI: 1.136 – 1.474, p=0.0001) and 32.1% (OR = 1.321, 95% CI: 1.169 – 1.493, p=0.0001), respectively. Regarding occupation, traders were about six (6) times more likely to be overweight/obese compared to the unemployed (OR: 5.727, 95% CI: 1.443–22.725, p=0.013). Other occupational categories did not show significant associations with any NCD risks. Education was inversely related to overweight/obesity; individuals with basic education had a reduced likelihood of being overweight/obese by approximately 52.6% (OR = 0.474, 95% CI: 0.244 – 0.918, p=0.027), secondary education by about 60.6% (OR = 0.394, 95% CI: 0.202 – 0.768, p=0.006), and above-secondary education by approximately 62.9% (OR = 0.371, 95% CI: 0.149 – 0.927, p=0.034) compared to those with no formal education; however, education did not significantly correlate with elevated blood glucose or blood pressure. Income level was significantly associated with all three NCD risks. Individuals earning < GH₵1000 had significantly lower odds of overweight/obesity (OR = 0.525, 95% CI: 0.296 – 0.933, p=0.028), while both lower income categories (< GH₵1000 and GH₵1000–1999) were associated with a reduced likelihood of elevated blood glucose (OR = 0.455, 95% CI: 0.260 – 0.797, p=0.006 and OR = 0.228, 95% CI: 0.100 – 0.518, p=0.0001 respectively) and elevated blood pressure (OR = 0.510, 95% CI: 0.268 – 0.967, p=0.039 and OR = 0.430, 95% CI: 0.253 – 0.732, p=0.002). Weight perception was significantly associated with overweight/obesity, with accurate perception markedly reducing the odds (OR = 0.087, 95% CI: 0.052 – 0.146, p<0.0001). However, no significant associations were observed between weight perception and elevated blood glucose or blood pressure.

**S1 Table:** **Bivariate Association between sociodemographic characteristics and NCD risks (overweight/obesity, elevated blood glucose, and elevated blood pressure) among the study participants**

| **Variable** | **^a^ Overweight/obesity** |  | **^b^ Elevated blood glucose** |  | **^c^ Elevated blood pressure** |
| --- | --- | --- | --- | --- | --- |
|  | OR (95% Cl) |  | OR (95% Cl) |  | OR (95% Cl) |
| **Age** | 1.061 (1.042, 1.081) * |  | 1.052 (1.030, 1.074) * |  | 1.053 (1.033, 1.073) * |
| **Occupation**  Ref= Unemployed  Professional/officer  Trader  Other | 1.000  4.167(0.998, 17.401)  5.727(1.443, 22.725) *  3.889(0.797, 18.975) |  | 1.000  2.323 (0.274, 19.698)  2.621 (0.326, 21.107)  0.818 (0.066, 10.196) |  | 1.000  1.866 (0.252, 13.811)  2.464 (0.349, 17.372)  1.479 (0.166, 13.169) |
| **Education status**  Ref= None  Basic  Secondary  Above | 1.000  0.474 (0.244, 0.918) *  0.394 (0.202, 0.768) *  0.371 (0.149, 0.927) * |  | 1.000  1.285 (0.668, 2.473)  0.688 (0.335, 1.412)  0.701 (0.234, 2.101) |  | 1.000  0.686 (0.384, 1.225)  0.556 (0.303, 1.019)  0.392 (0.144, 1.066) |
| **Parity** | 1.432 (1.242, 1.651) * |  | 1.294 (1.136, 1.474) * |  | 1.321 (1.169, 1.493) * |
| **Marital status**  Ref= Never married  Married/cohabiting  Previously married | 1.000  0.942 (0.593, 1.497)  1.160 (0.544, 2.473) |  | 1.000  1.044 (0.612, 1.781)  1.063 (0.458, 2.468) |  | 1.000  1.000 (0.620, 1.612)  1.104 (0.523, 2.333) |
| **^d^Income category**  Ref= ≥ 2000  < 1000  1000 - 1999 | 1.000  0.525 (0.296, 0.933) *  0.666 (0.337, 1.315) |  | 1.000  0.455 (0.260, 0.797) *  0.228 (0.100, 0.518) * |  | 1.000  0.510 (0.268, 0.967) *  0.430 (0.253, 0.732) * |
| **Weight perception**  Ref= Inaccurate  Accurate | 1.000  0.087 (0.052, 0.146) * |  | 1.000  0.580 (0.322, 1.045) |  | 1.000  0.836 (0.510, 1.369) |
| ^a^ Refers to body mass index which indicates overweight/obese i.e. BMI ≥ 25.0 kg/m^2^  ^b^ Refers to random blood glucose which indicates elevated blood glucose i.e. RBS ≥ 11.1 mmol/L  ^c^ Refers to blood pressure which indicates elevated blood pressure i.e. BP ≥ 140/90 mmHg  ^d^ Amount in Ghana Cedis (GH₵); represents monthly income  Ref.= Reference category,  *Statistically significant at p<0.05 | | | | | |
